# Neuroendocrine Neoplasms of the Gastrointestinal Tract: Morphology, WHO 2022 Grading, and Prognostic Perspectives

**DOI:** 10.7759/cureus.100913

**Published:** 2026-01-06

**Authors:** Hussein Qasim, Shaima' Dibian, Mohammad Abu Shugaer, Karis Khattab, Mudhaffer Touqan, Matteo Luigi Giuseppe Leoni, Giustino Varrassi

**Affiliations:** 1 Pathology and Laboratory Medicine, Jordan University of Science and Technology, Irbid, JOR; 2 Medicine, Jordan University of Science and Technology, Irbid, JOR; 3 Emergency Medicine, Azraq Refugee Camp Hospital International Medical Corps, Amman, JOR; 4 Medical and Surgical Sciences and Translational Medicine, Sapienza University, Rome, ITA; 5 Pain Medicine, Fondazione Paolo Procacci, Rome, ITA

**Keywords:** gastrointestinal neuroendocrine neoplasms, ki-67, minen, net g3, neuroendocrine carcinomas, neuroendocrine tumors, who 2022 classification

## Abstract

Gastrointestinal neuroendocrine neoplasms (GI NENs) represent a heterogeneous group of tumors distinguished by variable morphology, proliferative behavior, and clinical outcomes. Their incidence continues to rise globally, driven by improved detection methods and increased understanding of neuroendocrine biology. This narrative review synthesizes current knowledge on the morphologic, immunohistochemical, and molecular features of GI NENs across anatomic sites, highlighting advances in diagnostic evaluation and the prognostic significance of the WHO 2022 grade. Key diagnostic challenges, including differentiating neuroendocrine tumor grade 3 (NET G3) from neuroendocrine carcinoma (NEC) and accurately assessing mixed neuroendocrine-non-neuroendocrine neoplasms (MiNENs), are examined alongside sources of variability such as Ki-67 interpretation and sampling limitations. Emerging prognostic biomarkers, digital pathology applications, and evolving therapeutic strategies, particularly somatostatin analogs, peptide receptor radionuclide therapy, and systemic chemotherapy, are reviewed in the context of precision oncology. Continued integration of molecular profiling, artificial intelligence, and standardized diagnostic approaches promises to refine prognostication and personalize management for patients with GI NENs.

## Introduction and background

Neuroendocrine neoplasms (NENs) of the gastrointestinal (GI) tract represent a biologically diverse and increasingly recognized group of tumors arising from neuroendocrine cells dispersed throughout the mucosa and submucosa of the digestive system [1]. These cells, which play a critical role in regulating gut motility, secretion, and metabolic homeostasis, can give rise to neoplasms ranging from indolent, well-differentiated tumors to highly aggressive carcinomas with rapid progression and poor clinical outcomes [2]. Over the past two decades, the incidence of GI NENs has risen steadily worldwide, a trend attributed not only to evolving molecular insights and heightened clinical awareness but also to improvements in endoscopic detection, radiologic imaging, and histopathologic surveillance [3]. As a result, NENs now constitute one of the fastest-growing categories of GI malignancies in epidemiologic registries [4].

Historically referred to as “carcinoid tumors,” GI NENs were once viewed as largely benign or low-grade lesions. However, contemporary research has demonstrated that these neoplasms exhibit a wide spectrum of biological behavior driven by complex interactions among tumor differentiation, proliferative activity, anatomic site, and underlying genomic alterations [5]. This growing understanding has necessitated more refined classification systems capable of capturing the nuances of tumor morphology and clinical behavior [6]. The World Health Organization (WHO) has played a central role in shaping these frameworks, culminating most recently in the WHO 2022 classification, which provides a standardized, multidisciplinary approach for diagnosing and grading GI NENs [7]. 

The WHO 2022 updates underscore the essential role of proliferation-based grading, incorporating mitotic rate and Ki-67 index to stratify well-differentiated neuroendocrine tumors (NETs) into Grades 1, 2, and 3 [8]. This refinement addresses a long-standing challenge in GI neuroendocrine pathology: the recognition that high-grade morphology does not necessarily equate to poor differentiation [9]. Indeed, well-differentiated Grade 3 NETs are now recognized as distinct from neuroendocrine carcinomas (NECs), reflecting their unique clinical course and management considerations [10]. These changes have significant diagnostic and prognostic implications, influencing decisions ranging from surgical intervention to systemic therapy and peptide receptor radionuclide therapy (PRRT) [10]. A particularly important refinement in the WHO 2022 classification is the formal recognition of well-differentiated Grade 3 NETs as a distinct biological entity, separating them from poorly differentiated NECs despite overlapping proliferative indices [10].

Given this evolving landscape, a comprehensive review synthesizing current understanding of morphology, WHO 2022 grading, and prognostic perspectives is timely and necessary. This narrative review aims to describe the key morphologic and immunohistochemical hallmarks of GI NENs across different anatomic sites, summarize and clarify the updated WHO 2022 diagnostic and grading framework, and examine the prognostic factors influencing clinical outcomes, including emerging biomarkers and therapeutic implications.

## Review

Methods

This narrative review was developed to synthesize current knowledge regarding the morphology, grading, diagnostic evaluation, and prognostic implications of gastrointestinal neuroendocrine neoplasms (GI NENs), with a particular focus on the updates introduced in the 2022 World Health Organization (WHO) classification. A comprehensive search of the literature was conducted using PubMed, Scopus, Web of Science, and Google Scholar, covering publications from 2000 to 2025. Search terms included combinations of “gastrointestinal neuroendocrine tumors,” “neuroendocrine neoplasms,” “NET G3,” “neuroendocrine carcinoma,” “MiNEN,” “WHO classification,” “Ki-67,” “grading,” “prognosis,” and “somatostatin receptor.” Additional sources were identified by manually reviewing the reference lists of selected articles and relevant contemporary review papers. Studies were included if they presented original data or comprehensive analyses pertaining to GI NEN morphology, immunohistochemistry, molecular alterations, or prognostic features and if they contributed directly to the current understanding of WHO 2017-2022 classification refinements. Only peer-reviewed English-language publications were considered. Studies were excluded if they lacked sufficient methodological detail, were primarily conference abstracts, or did not address GI NEN classification or biology.

Titles and abstracts were screened for relevance, followed by full-text evaluation of eligible studies. Information was extracted regarding morphologic patterns, immunohistochemical markers, Ki-67 assessment methods, molecular signatures, site-specific behavior, and factors influencing prognosis and treatment. Emphasis was placed on literature comparing well-differentiated NET G3 with poorly differentiated NECs, as well as studies addressing diagnostic challenges such as sampling variability and interobserver differences in Ki-67 quantification. Evidence from high-quality studies, expert consensus guidelines, and recent WHO classification documents was prioritized when synthesizing conclusions. Due to substantial heterogeneity across study designs and outcome measures, no meta-analysis was performed. Instead, findings were integrated narratively to highlight converging themes, evolving diagnostic frameworks, and areas of ongoing controversy. As the study utilized previously published data, institutional review board approval and informed consent were not required.

Classification of gastrointestinal neuroendocrine neoplasms

Historical Evolution of Classification

The classification of GI NENs has evolved substantially over the past century [11]. The earliest descriptor, introduced by Oberndorfer in 1907, labeled these tumors as “carcinoid,” implying a benign or less malignant nature compared with typical GI adenocarcinomas [12]. While this term persisted for decades, it eventually became clear that these neoplasms displayed a wide spectrum of biological behaviors, ranging from indolent to highly aggressive [13]. Improvements in immunohistochemistry, particularly the introduction of chromogranin A and synaptophysin staining, further refined the ability to detect neuroendocrine differentiation and contributed to a more accurate understanding of these tumors [14].

Major conceptual progress occurred with the WHO 2000 and WHO 2010 classifications, which introduced a grading system based on proliferative activity using mitotic rate and Ki-67 index [15]. These updates recognized that proliferation, rather than morphology alone, carried significant prognostic value [15]. However, clinical experience soon revealed a critical gap: some tumors with well-differentiated morphology exhibited high proliferation rates but behaved distinctly from poorly differentiated NECs [16]. This discrepancy prompted refinement in subsequent classifications. The WHO 2017 and WHO 2019 updates formally addressed this issue by defining a new category, well-differentiated NET G3 [17]. This addition acknowledged that high-grade proliferation does not necessarily indicate poor differentiation and clarified the distinction between NET G3 and NEC [17]. Building on these iterations, the WHO 2022 classification provides the most integrated and biologically informed framework to date, incorporating both morphologic and molecular criteria to create a unified system applicable across the entire GI tract [18].

WHO 2022 Classification Overview

The WHO 2022 classification organizes gastrointestinal neuroendocrine neoplasms into three major categories: well-differentiated neuroendocrine tumors (NETs), poorly differentiated neuroendocrine carcinomas (NECs), and mixed neuroendocrine-non-neuroendocrine neoplasms (MiNENs) [7]. Well-differentiated NETs maintain organoid architecture, uniform cytologic features, and strong expression of neuroendocrine markers [5]. They are graded (G1-G3) based on Ki-67 index and mitotic activity [19]. Importantly, NET G3 is now recognized as a distinct entity that retains well-differentiated morphology despite a high proliferation rate. These tumors behave differently from NECs, demonstrating more favorable outcomes and responsiveness to therapies such as somatostatin analogs and peptide receptor radionuclide therapy (PRRT) [20]. Neuroendocrine carcinomas (NECs) represent the poorly differentiated end of the spectrum and include small-cell and large-cell phenotypes [21]. They are characterized by marked nuclear atypia, brisk mitotic activity, extensive necrosis, and loss of typical neuroendocrine architectural patterns [16].

Neuroendocrine carcinomas typically exhibit molecular features shared with high-grade carcinomas, most notably alterations in *TP53 *and *RB1 *[22]. Clinically, they are highly aggressive tumors requiring systemic cytotoxic chemotherapy, often similar to regimens used for small-cell lung carcinoma [23]. MiNENs contain both a neuroendocrine and a non-neuroendocrine component, each comprising at least 30% of the tumor [24]. The behavior of these tumors depends largely on the most aggressive component, and their molecular profiles reflect their dual differentiation pathways [24]. MiNENs pose diagnostic challenges due to sampling variability and the need for careful morphologic and immunohistochemical evaluation [24]. A key strength of the WHO 2022 classification lies in its emphasis on the distinct genetic and biological features that differentiate NETs from NECs [7]. NETs frequently harbor alterations in genes such as *MEN1*, *DAXX*, and *ATRX*, supporting their origin from well-differentiated neuroendocrine cell lineages [25]. In contrast, NECs more commonly demonstrate mutations in *TP53 *and *RB1*, confirming their alignment with high-grade carcinomas rather than with typical neuroendocrine tumors [26].

Understanding these molecular distinctions is essential because they correlate strongly with therapeutic response and prognosis. From a conceptual standpoint, the WHO framework underscores that differentiation, not proliferation, is the primary determinant of tumor classification [27]. Well-differentiated NETs and poorly differentiated NECs are biologically distinct diseases, even when their proliferation indices overlap [28]. The recognition of NET G3 further reinforces this principle by separating well-differentiated high-grade tumors from carcinomas that are fundamentally dedifferentiated [29]. This clear delineation enhances diagnostic accuracy, improves prognostic stratification, and guides appropriate treatment selection.

Morphologic features of gastrointestinal neuroendocrine neoplasms

General Histologic Hallmarks

Gastrointestinal neuroendocrine neoplasms share a set of defining morphologic features that reflect their origin from neuroendocrine cells of the diffuse endocrine system [30]. Well-differentiated NETs typically demonstrate organoid architecture, including nested, trabecular, gyriform, or rosette-like patterns [31]. The neoplastic cells usually have round to oval nuclei with finely granular “salt-and-pepper” chromatin, a hallmark of neuroendocrine differentiation [32]. Cytoplasm is moderate and eosinophilic, and cell borders tend to be indistinct [33]. Although mitotic activity is generally low in NETs, distinctions in proliferation are essential for grading and prognostication. Immunohistochemistry (IHC) plays a central role in confirming neuroendocrine differentiation [34]. The two most commonly expressed markers are synaptophysin, a sensitive marker of neuroendocrine vesicles, and chromogranin A, which is more specific but may show weaker expression in high-grade tumors [35]. INSM1 has emerged as an additional sensitive nuclear marker that helps reinforce the neuroendocrine lineage [36]. Assessment of Ki-67 proliferation index is mandatory for WHO grading, providing a quantitative measure of proliferative activity that distinguishes low-grade from high-grade lesions [37].

Site-Specific Morphology

Esophageal NENs are exceedingly rare and are overwhelmingly represented by poorly differentiated NECs rather than well-differentiated NETs [38]. These tumors typically exhibit small-cell or large-cell morphology with high mitotic rates, prominent necrosis, and marked nuclear atypia [38]. Small-cell NECs demonstrate molding nuclei, scant cytoplasm, and diffuse chromatin, while large-cell NECs display more abundant cytoplasm and vesicular nuclei with prominent nucleoli [39]. Clinically, esophageal NECs exhibit aggressive behavior with early metastasis and poor overall prognosis, mirroring small-cell lung carcinoma both morphologically and biologically [40]. Gastric NETs encompass a heterogeneous group categorized into three subtypes, each with distinct pathophysiology and morphology [41].

Type I gastric NETs arise in the setting of chronic autoimmune atrophic gastritis and hypergastrinemia [42]. They are typically small, multifocal, and confined to the mucosa or submucosa. The background mucosa often shows enterochromaffin-like (ECL) cell hyperplasia [43]. Type II gastric NETs occur in the context of gastrinoma and Zollinger-Ellison syndrome, often associated with *MEN1*. These tumors resemble Type I lesions morphologically but tend to be larger and more clinically aggressive [44]. Moreover, Type III gastric NETs are sporadic lesions unrelated to hypergastrinemia [45]. They are solitary, larger, invade deeper layers, and exhibit higher proliferative activity [45]. Type III tumors may display more pronounced cytologic atypia and have a greater propensity for metastasis [45]. Poorly differentiated gastric NECs also arise but are biologically distinct, demonstrating high-grade cytologic features and lacking the organoid patterns typical of NETs [46]. Their aggressive behavior necessitates classification and treatment distinct from those of gastric NETs [46].

Small intestinal NETs, especially those arising in the ileum, exhibit some of the most classic morphologic features of well-differentiated neuroendocrine tumors [47]. They typically form nests or trabeculae of uniform cells with salt-and-pepper chromatin and minimal atypia [48]. A hallmark of midgut NETs is their functional capacity for serotonin production, which can lead to mesenteric fibrosis and desmoplastic reactions [49]. These tumors often induce marked retractile fibrosis around the mesenteric root, sometimes causing bowel obstruction or ischemia [49]. Midgut NETs frequently metastasize to regional lymph nodes and the liver, sometimes even when the primary tumor is small and clinically silent [50]. Metastatic lesions often retain organoid architecture, although fibrotic stroma may be more prominent [50]. Their relatively indolent growth contrasts with their propensity for widespread dissemination [50].

Appendiceal NETs are typically discovered incidentally during appendectomy. They most often arise at the distal tip of the appendix and are usually small, well-differentiated tumors with a very favorable prognosis [51]. Morphologically, they display classic NET features, including nests or ribbons of uniform cells with granular chromatin [52]. Subserosal involvement may occur, but lymph node metastasis is rare for tumors smaller than 2 cm [53]. A unique subset of appendiceal NENs includes goblet cell adenocarcinomas (formerly “goblet cell carcinoids”), which show mixed mucinous and neuroendocrine differentiation [54]. These lesions are more aggressive than typical appendiceal NETs and behave more like adenocarcinomas [55]. Their biphasic morphology, with mucin-containing cells and neuroendocrine-like components, necessitates careful histopathologic evaluation and distinction from conventional NETs [56].

Colorectal NENs encompass a spectrum ranging from well-differentiated NETs to highly aggressive NECs [57]. Rectal NETs are more common than colonic NETs and typically present as small, submucosal nodules detected during endoscopy [58]. They show classic neuroendocrine features, often with less pronounced fibrosis compared with small intestinal NETs [59]. Despite their generally favorable prognosis, larger rectal NETs (>2 cm) may exhibit higher rates of metastasis [58]. In contrast, colonic NENs are more often poorly differentiated NECs with aggressive behavior, high mitotic activity, extensive necrosis, and frequent metastasis at diagnosis [60]. The distinction between high-grade NET G3 and NEC is particularly important in the colon, as NET G3 tumors retain well-differentiated features and have different molecular drivers and treatment responses [61]. NECs, dominated by TP53 and RB1 alterations, behave similarly to high-grade non-small cell carcinomas [62]. Although pancreatoduodenal NENs are sometimes considered separately due to their foregut origin, their morphologic spectrum parallels that of GI NENs [63].

**Figure 1:**
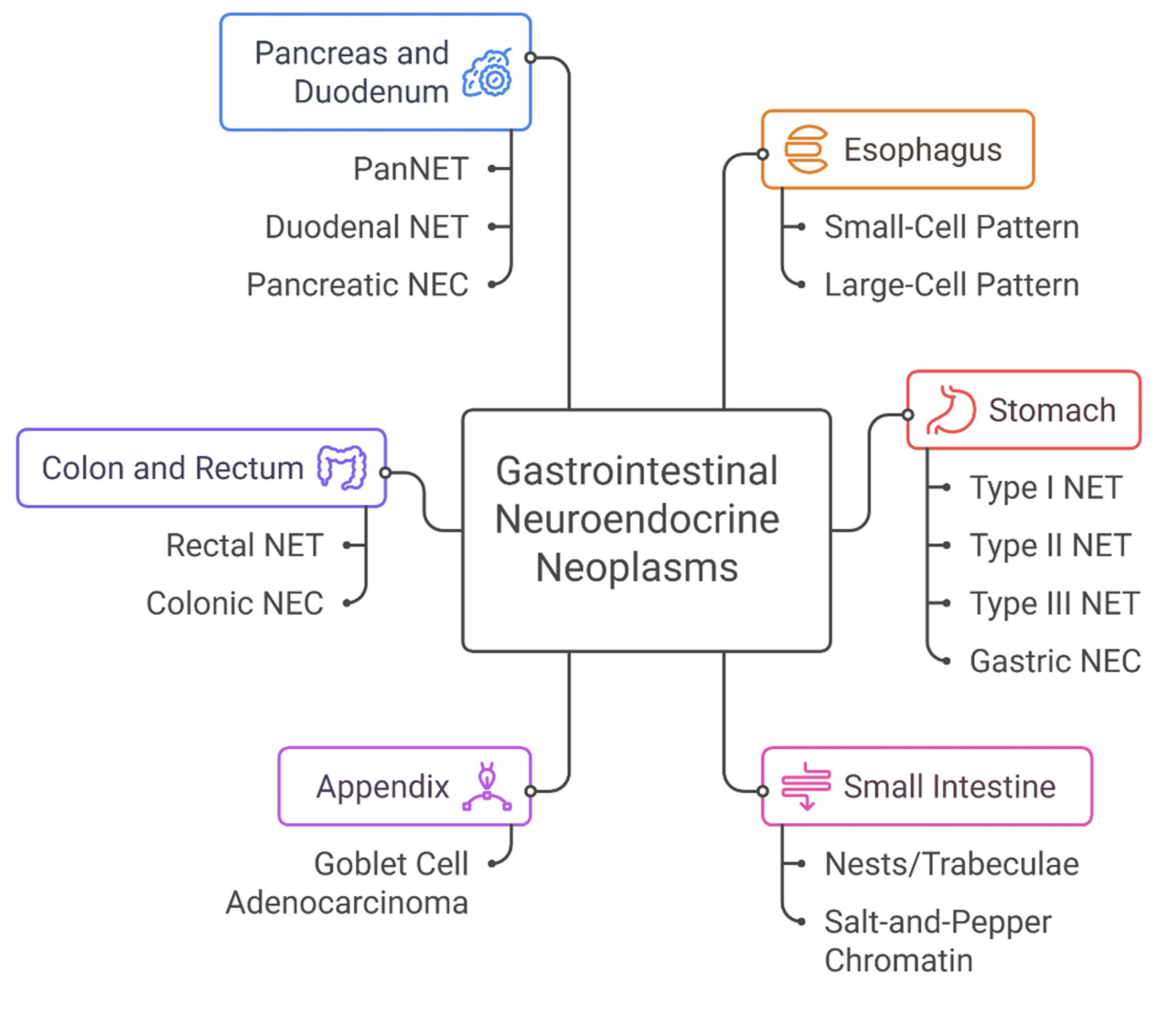
Summary of site-specific morphologies of gastrointestinal neuroendocrine neoplasms Image is original and created by Dr. Karis Khattab on Microsoft PowerPoint (Microsoft Corporation, Redmond, Washington, United States). PanNET, pancreatic neuroendocrine tumor; NET, neuroendocrine tumor; NEC, neuroendocrine carcinoma.

Well-differentiated pancreatic NETs (PanNETs) show organoid patterns and may have distinctive hormonal syndromes depending on peptide secretion (e.g., insulinoma, gastrinoma) [64]. Mutations in *MEN1*, *DAXX*, and *ATRX *are characteristic [65]. Poorly differentiated pancreatic NECs resemble NECs from other sites and carry a dismal prognosis [65]. Duodenal NETs are often gastrin-producing (Type II gastric NET association) and exhibit small, uniform cells with minimal atypia [66]. Their behavior varies by size and depth but tends to be less aggressive than pancreatic NECs [66]. Figure 1 shows a summary of site-specific morphologies of gastrointestinal neuroendocrine neoplasms.

World Health Organization 2022 grading system

Rationale for Grading

Grading plays a central role in the modern classification of gastrointestinal neuroendocrine neoplasms, providing an objective measure of biological aggressiveness and prognostic potential [67]. The WHO 2022 system incorporates two key proliferation metrics, mitotic count and Ki-67 labeling index, to stratify NETs into clinically meaningful categories [19]. Numerous studies have demonstrated a strong correlation between proliferative activity and patient outcomes: tumors with low mitotic rates and low Ki-67 indices typically follow an indolent course, whereas those with elevated proliferation exhibit more rapid growth, greater metastatic potential, and significantly reduced survival [68]. Importantly, grading complements morphologic assessment by identifying biologically aggressive tumors that may otherwise appear deceptively bland under routine microscopy [69]. Thus, proliferation-based grading is essential for guiding therapeutic strategies, determining surveillance intervals, and predicting long-term prognosis.

Grading of Well-Differentiated Neuroendocrine Tumors (NETs)

**Figure 2:**
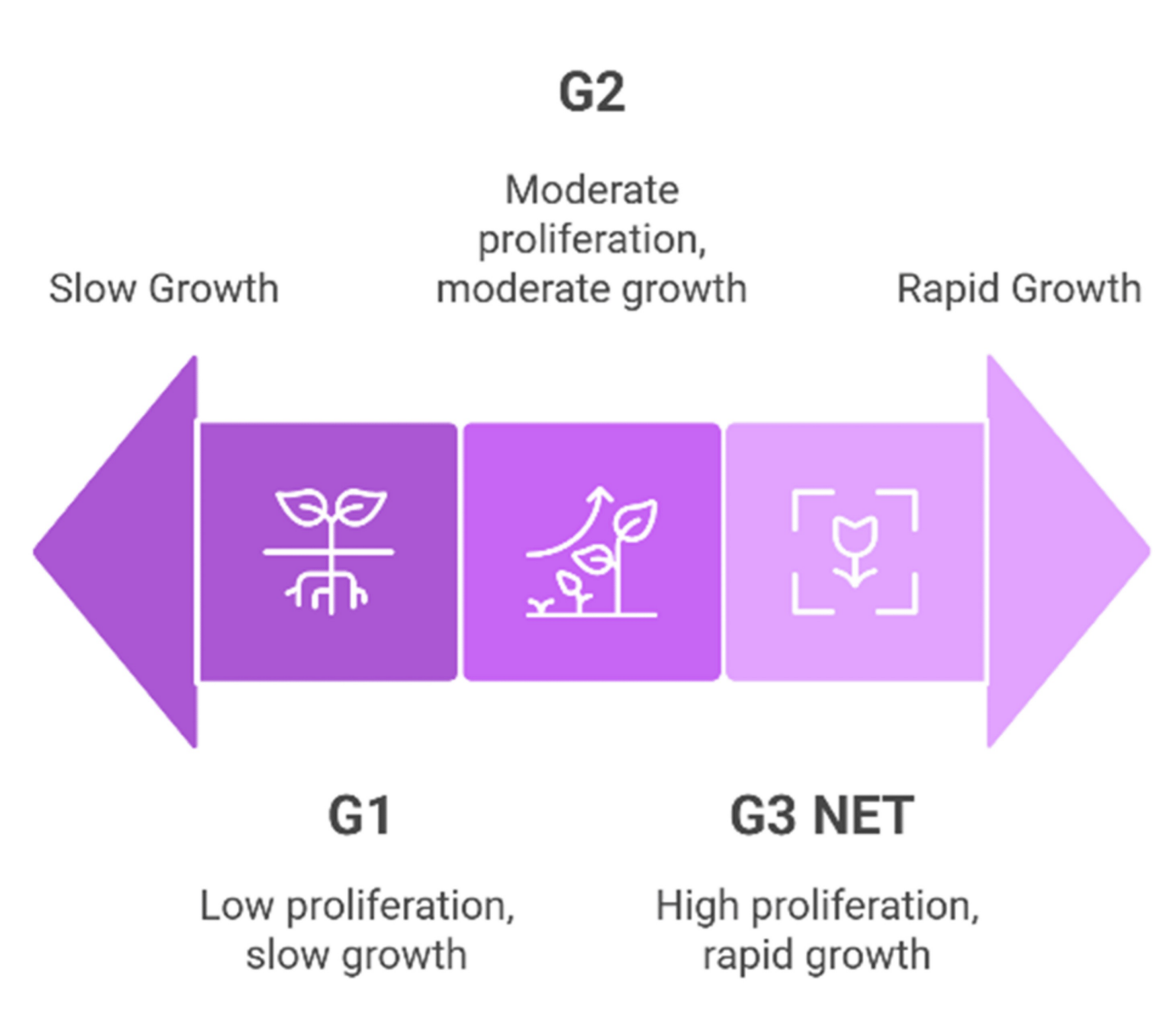
WHO 2022 classification stratification of well-differentiated NETs based on Ki-67 proliferation index and mitotic activity Image is original and created by Dr. Karis Khattab on Microsoft PowerPoint (Microsoft Corporation, Redmond, Washington, United States). G1, grade 1; G2, grade 2; G3 NET, grade 3 neuroendocrine tumor.

As shown in Figure 2, the WHO 2022 classification stratifies well-differentiated NETs into three grades based on their Ki-67 proliferation index and mitotic activity: Grade 1 (G1): Ki-67 index <3% and mitotic rate <2 per 2 mm²; Grade 2 (G2): Ki-67 index 3-20% or mitotic rate 2-20 per 2 mm²; Grade 3 (G3 NET): Ki-67 index >20% and/or mitotic rate >20 per 2 mm², while maintaining well-differentiated morphology

The recognition of well-differentiated G3 NETs represents a pivotal advancement in neuroendocrine pathology [70]. Prior to their formal classification, high-proliferation tumors were often misinterpreted as NECs, leading to inappropriate management approaches [71]. Morphologically, G3 NETs retain organoid architecture, cytologic uniformity, and strong neuroendocrine marker expression, distinguishing them from the overt atypia and architectural loss characteristic of NECs [71]. Clinically, G3 NETs demonstrate intermediate behavior: they are more aggressive than NET G1 and G2 but significantly less aggressive than NECs [72]. This distinction has substantial therapeutic implications, as G3 NETs typically respond better to somatostatin analogs, targeted therapies, and peptide receptor radionuclide therapy (PRRT) than to platinum-based chemotherapy, which is more effective in NECs [72]. The clear separation of NET G3 from NEC in the WHO 2022 system ensures more accurate prognostication and treatment stratification, reflecting a biologically grounded approach to grading.

Grading and classification of neuroendocrine carcinomas (NECs)

Neuroendocrine carcinomas represent the high-grade, poorly differentiated end of the neuroendocrine neoplasm spectrum [73]. Unlike NETs, NECs are not graded using Ki-67 thresholds, because their high proliferation is a defining feature rather than a variable parameter [74]. NECs are instead categorized based on their morphology into small-cell and large-cell subtypes [75]. Small-cell NECs consist of small, round to fusiform cells with hyperchromatic nuclei, inconspicuous nucleoli, scant cytoplasm, nuclear molding, and frequent mitoses [76]. Large-cell NECs display more abundant cytoplasm, vesicular nuclei, and prominent nucleoli but remain poorly differentiated with diffuse growth patterns and extensive necrosis [77]. A critical distinction between NECs and well-differentiated tumors lies in their molecular profile [78]. NECs characteristically harbor *TP53 *and *RB1 *gene alterations, reflecting a pathway of carcinogenesis shared with other high-grade epithelial malignancies, particularly small-cell lung carcinoma [79]. These molecular signatures reinforce the concept that NECs arise through dedifferentiation rather than progression from well-differentiated NETs [80]. Their loss of typical neuroendocrine architecture, combined with marked cytologic atypia and high proliferation, results in a uniformly aggressive clinical course requiring cytotoxic chemotherapy [80]. Thus, the classification of NECs within the WHO 2022 system rests on morphologic and molecular features rather than proliferative thresholds, distinguishing them clearly from high-grade NETs and guiding appropriate treatment choices.

Grading principles in mixed neuroendocrine-non-neuroendocrine neoplasms (MiNENs)

Mixed neuroendocrine-non-neuroendocrine neoplasms (MiNENs) are defined by the presence of both a neuroendocrine and a non-neuroendocrine component, each comprising at least 30% of the tumor [81]. Grading MiNENs poses unique challenges because the two components often display markedly different morphologic and biological behaviors [82]. In practice, each component should be evaluated and graded independently according to established WHO criteria for its respective lineage [83]. Prognosis is typically driven by the more aggressive component, which in many cases is the neuroendocrine portion, particularly when it exhibits high-grade features consistent with NEC [21]. A major challenge in MiNEN classification arises from sampling variability [84]. Limited biopsy samples may capture only one component, resulting in underdiagnosis or misclassification [84]. Comprehensive histologic evaluation, including thorough sectioning of resected specimens and judicious use of immunohistochemistry, is essential for accurate recognition [85]. Reporting should explicitly describe the proportion, grade, and differentiation of each component to guide clinical decision-making.

Diagnostic approach

Pathologic Evaluation Workflow

The diagnostic evaluation of gastrointestinal neuroendocrine neoplasms relies on a systematic and meticulous pathologic workflow that integrates gross examination, microscopic morphology, immunohistochemistry, and molecular analysis [86]. Gross inspection should include careful assessment of tumor size, location, depth of invasion, and relationship to surgical margins [86]. Adequate sampling is essential, as some lesions, particularly MiNENs, may exhibit heterogeneous architecture that is not apparent on limited biopsy material [87]. For small biopsy specimens, pathologists must be cautious when interpreting limited tissue, ensuring that necrotic or crushed regions do not obscure diagnostic features. Microscopically, the first step involves identifying architectural patterns and cytologic features characteristic of neuroendocrine differentiation [88]. Well-differentiated NETs typically display organoid patterns such as nests, trabeculae, rosettes, or ribbons, along with uniform nuclei bearing finely stippled “salt-and-pepper” chromatin [89]. The presence or absence of necrosis, mitotic activity, cytologic pleomorphism, and architectural disorganization provides early clues about tumor grade and differentiation [5]. Poorly differentiated neuroendocrine carcinomas (NECs) exhibit diffuse sheets of cells, marked atypia, brisk mitotic activity, and extensive necrosis, features that sharply contrast with the more orderly appearance of NETs [5].

Immunohistochemical Algorithms

Immunohistochemistry (IHC) plays a central role in confirming neuroendocrine differentiation, assessing proliferation, and determining the site of origin [90]. The three core neuroendocrine markers, synaptophysin, chromogranin A, and INSM1, form the foundation of most diagnostic algorithms [36]. Synaptophysin is highly sensitive and stains the majority of neuroendocrine tumors, including poorly differentiated NECs [91]. Chromogranin A is more specific but may show weaker or patchy expression in high-grade tumors [91]. INSM1, a transcription factor, has emerged as a robust nuclear marker with high sensitivity and specificity across both well-differentiated NETs and NECs [91]. Assessment of the Ki-67 proliferation index is essential for grading according to the WHO 2022 criteria [92]. Accurate determination requires counting at least 500-2,000 tumor cells in the “hot spot” areas with the highest labeling density [93]. Pitfalls include sampling bias, interobserver variability, underestimation due to crush artifact, and overestimation caused by inflammatory infiltrates or proliferating entrapped non-neoplastic cells [94]. For this reason, Ki-67 interpretation should always be paired with morphologic assessment, particularly when distinguishing NET G3 from NEC [95]. A panel of additional markers may be used for site attribution, especially in metastatic disease where the primary location is uncertain [96]. Cytokeratin (CK) profiles help confirm epithelial origin [97]. CDX2, a transcription factor expressed in intestinal epithelium, supports a midgut origin when positive [98]. ISL1 and PAX8 are frequently expressed in pancreatic and some upper GI neuroendocrine tumors, aiding in differentiation from colorectal primaries [99]. Together, these markers support a lineage-specific diagnostic approach, enabling more accurate classification and appropriate treatment strategies.

Molecular and Genetic Testing

Molecular testing increasingly complements histologic and immunophenotypic evaluation, particularly in ambiguous or high-grade cases [100]. In well-differentiated NETs, mutations in *MEN1*, *DAXX*, and *ATRX *are commonly observed and provide insight into tumor pathogenesis [101]. Loss of *DAXX*/*ATRX *expression by immunohistochemistry can serve as a surrogate for underlying genetic alterations and has been associated with distinct clinical behavior and outcomes, especially in pancreatic NETs [101]. Although these mutations do not currently dictate targeted therapy, their presence supports the diagnosis of a well-differentiated NET and helps distinguish NETs from NECs [102]. In contrast, poorly differentiated NECs often harbor alterations in the *TP53 *and *RB1 *pathways, aligning them molecularly with high-grade non-neuroendocrine carcinomas [62]. Identifying these mutations through next-generation sequencing (NGS) can help confirm the diagnosis in challenging cases, particularly when morphology and Ki-67 index alone create ambiguity between NET G3 and NEC [103]. NGS also aids in identifying mismatch repair deficiency or other actionable alterations that may influence therapeutic decisions [104]. Emerging molecular biomarkers, including somatostatin receptor expression (SSTR2), circulating tumor DNA, and transcriptomic signatures, hold promise for improving prognostication and individualized treatment [105]. While not yet incorporated into routine diagnostic workflows for all GI NENs, these advances suggest a growing role for molecular precision tools in the future classification and management of neuroendocrine neoplasia [105].

Prognostic perspectives

Impact of the WHO 2022 Grade on Survival

The WHO 2022 grading system plays a central role in predicting clinical outcomes for GI NENs [7]. Numerous studies have demonstrated clear distinctions in survival across the NET G1, G2, and G3 categories, with grade serving as one of the strongest independent prognostic indicators [61]. NET G1 tumors, characterized by low mitotic rates and Ki-67 indices <3%, typically exhibit very favorable survival, often exceeding a decade even in the presence of metastatic disease [106]. NET G2 tumors, with intermediate proliferation (Ki-67 3-20%), display more variable clinical behavior but generally maintain a better prognosis than high-grade neoplasms [74]. The introduction of well-differentiated NET G3 as a distinct category has refined prognostic stratification by separating these tumors from NECs [29]. Although NET G3 tumors exhibit high proliferative indices (Ki-67 >20%), they retain well-differentiated morphology and follow a less aggressive clinical course than NECs [61]. Survival curves demonstrate that NET G3 tumors fall between NET G2 and NEC, emphasizing the importance of differentiating high-grade NETs from poorly differentiated carcinomas [72]. NECs, regardless of small-cell or large-cell morphology, show uniformly poor survival with rapid progression and limited responsiveness to non-cytotoxic therapies [21]. Thus, the WHO grade integrates proliferative rate with morphology and remains a powerful predictor of long-term survival.

Site-Specific Prognosis

The anatomic origin of GI NENs significantly influences their prognosis, even when controlling for tumor grade [67]. Small intestinal (midgut) NETs often follow an indolent but metastatic course; despite frequent regional or hepatic metastasis at diagnosis, survival rates remain high due to their slow-growing nature and strong responsiveness to somatostatin-based therapies [107]. Appendiceal NETs, especially those <2 cm, carry an excellent prognosis with rare nodal involvement and minimal risk of distant spread [51]. Their favorable outcomes reflect their low-grade nature and frequent incidental discovery [51]. In contrast, colonic NENs are often diagnosed at advanced stages and are more likely to be poorly differentiated NECs [108]. These tumors are associated with significantly worse outcomes compared with NETs of the rectum or small intestine [109]. Rectal NETs, although common, are usually small, low-grade, and highly amenable to endoscopic removal, resulting in an excellent prognosis for most patients [110]. Gastric NENs demonstrate diverse prognoses depending on subtype: Type I lesions (associated with autoimmune atrophic gastritis) have very favorable outcomes, whereas Type III gastric NETs, which are sporadic and not driven by hypergastrinemia, tend to behave more aggressively and possess a higher metastatic potential [111]. These site-specific variations underscore the need for an integrated diagnostic approach that accounts for anatomic origin, underlying pathophysiology, and tumor grade.

Emerging Prognostic Markers

Beyond traditional morphologic grading, several emerging biomarkers offer additional prognostic insight for GI NENs [112]. The tumor microenvironment, including stromal composition, immune infiltration, and angiogenic activity, has been increasingly recognized as a determinant of tumor behavior and response to therapy [113]. Small intestinal NETs, for example, often induce a desmoplastic stromal reaction, which may influence local complications and metastatic patterns [114]. Somatostatin receptor (SSTR2) expression, detectable by immunohistochemistry or functional imaging (e.g., Ga-68 DOTATATE PET), is a valuable prognostic and predictive biomarker [115]. Strong SSTR2 expression correlates with well-differentiated morphology and favorable response to somatostatin analogs and peptide receptor radionuclide therapy (PRRT) [116]. In contrast, NECs typically lack meaningful SSTR expression, limiting the therapeutic applicability of receptor-targeted approaches [117]. Chromogranin A, although affected by multiple confounders, remains a widely used marker for disease burden and recurrence monitoring [118]. More recently, multigene expression signatures such as the NETest have demonstrated promise for predicting treatment response, detecting early progression, and refining risk stratification [119]. These emerging markers collectively contribute to a more nuanced understanding of tumor biology and may soon complement existing grading frameworks in routine practice.

Treatment implications

Prognostic classification directly informs therapeutic decision-making in GI NENs [120]. Surgical management offers excellent outcomes for low-grade NETs, particularly those of the small intestine, appendix, and rectum, where complete resection is often curative for localized disease [121]. Higher-grade NETs (G2 and G3) may still benefit from surgical debulking when feasible, especially in cases of hepatic metastasis, given their relatively indolent growth and sensitivity to hormonal therapies [122]. Somatostatin analogs (SSAs) play a foundational role in controlling both hormonal symptoms and tumor growth in well-differentiated NETs, with demonstrated progression-free survival benefits [123]. The efficacy of SSAs is closely linked to tumor grade and SSTR expression, further tying prognosis to therapeutic strategy [123]. Peptide receptor radionuclide therapy (PRRT) has emerged as a highly effective modality for metastatic NETs with strong SSTR expression, particularly NETs of small intestinal origin [123]. NET G3 tumors may also respond to PRRT, although outcomes vary depending on Ki-67 index and differentiation status [124]. NECs, by contrast, exhibit limited benefit due to low receptor expression [124]. For poorly differentiated NECs, platinum-based chemotherapy remains the cornerstone of treatment [125]. Response rates are high initially, but remissions are often short-lived, reflecting the aggressive nature of these neoplasms [126]. Understanding the prognostic differences between NET G3 and NEC ensures that patients receive appropriate therapy, avoiding overly aggressive cytotoxic regimens in NET G3 while ensuring timely intervention in NEC [127].

Challenges and controversies

Despite significant refinements introduced by the WHO 2022 classification system, several challenges and controversies remain in the diagnostic and clinical evaluation of gastrointestinal neuroendocrine neoplasms (GI NENs) [17]. These challenges reflect both the inherent biological complexity of these tumors and the practical limitations of current diagnostic tools [17]. One of the most prominent diagnostic dilemmas involves distinguishing well-differentiated NET G3 from poorly differentiated NECs [128]. Although both categories exhibit high proliferative activity, their underlying biology, clinical behavior, and treatment responsiveness differ markedly [129]. NET G3 tumors retain organoid architecture and cytologic uniformity, whereas NECs display marked atypia, extensive necrosis, and molecular aberrations involving *TP53 *and *RB1 *[62]. However, these distinctions are not always clear on limited biopsy samples, especially when crush artifact, necrosis, or sampling bias obscures morphologic features [88].

Misclassification can lead to inappropriate management, overly aggressive chemotherapy in NET G3 or insufficient cytotoxic therapy in NEC, making accurate distinction critically important yet sometimes difficult [21]. A second area of controversy arises in the classification of MiNENs [83]. These tumors require each component to constitute at least 30% of the lesion; however, this threshold is arbitrary and does not necessarily reflect biological behavior [83]. Some tumors with a small but highly aggressive neuroendocrine component may behave more like NECs than adenocarcinomas, challenging the utility of strict percentage-based definitions [130]. The heterogeneity of MiNENs further complicates management, as treatment strategies must be tailored to the most aggressive component, yet identifying that component is not always straightforward [131].

Another significant challenge is the variability in Ki-67 assessment, which directly impacts tumor grading and subsequent prognostic and therapeutic decisions [131]. Interobserver variability, differences in counting methods, and heterogeneity of Ki-67 distribution within tumors all contribute to inconsistent proliferation measurements [132]. Hot-spot selection can vary between pathologists, potentially shifting a tumor across grading thresholds [133]. Automated digital image analysis may offer improved reproducibility in the future, but its widespread implementation remains limited [134]. Until standardized protocols are universally adopted, Ki-67 variability will continue to pose a challenge in ensuring reliable and reproducible grading [67]. Finally, limitations in tissue sampling represent a persistent obstacle in the accurate classification of GI NENs [135]. Small biopsy specimens may not capture the full morphologic spectrum of the tumor, particularly in heterogeneous neoplasms such as MiNENs or NECs [136]. Sampling error is especially problematic when necrotic areas predominate or when tumor cells are sparsely distributed within dense desmoplastic stroma [137]. In such cases, immunohistochemical and molecular testing may be inconclusive or misleading.

Future directions

Emerging technologies and expanding molecular insights are poised to significantly refine the diagnosis and management of gastrointestinal neuroendocrine neoplasms [138]. Increasingly, molecular profiling is expected to become part of routine classification, helping to distinguish well-differentiated NETs from NECs and identifying actionable alterations that may guide targeted therapies [139]. As genomic and epigenetic signatures become better defined, these tools will likely complement traditional morphology and proliferation-based grading [140]. Advances in digital pathology and artificial intelligence (AI) offer the potential to enhance diagnostic accuracy, particularly in assessing Ki-67 proliferation indices and distinguishing subtle morphologic differences between NET G3 and NEC [141]. AI-assisted algorithms may also help standardize interpretation, reducing interobserver variability and improving reproducibility across institutions [142]. Finally, ongoing research into emerging therapeutic targets is expanding the treatment landscape [143]. These include agents targeting DNA repair pathways, angiogenesis, metabolic vulnerabilities, and immune modulation [143]. As the molecular underpinnings of GI NENs become clearer, therapies tailored to specific genetic or phenotypic subgroups may further improve outcomes.

## Conclusions

Gastrointestinal neuroendocrine neoplasms encompass a broad spectrum of tumors with distinct morphologic and biological behaviors. The WHO 2022 classification has greatly improved diagnostic precision by emphasizing differentiation, proliferation, and molecular features, allowing clearer separation of well-differentiated NETs from aggressive NECs. Morphology, immunohistochemistry, and Ki-67 assessment remain foundational tools, increasingly supported by molecular profiling to refine classification and guide treatment. Despite these advances, challenges persist, particularly in distinguishing NET G3 from NEC, evaluating heterogeneous tumors such as MiNENs, and interpreting Ki-67 with consistency. Ongoing developments in digital pathology, artificial intelligence, and molecular diagnostics promise to address many of these limitations and enhance prognostic accuracy. Overall, integrating morphology with modern molecular and computational tools will continue to advance the understanding and management of GI NENs, enabling more personalized and effective therapeutic strategies for patients.
